# Integration of gender-transformative interventions into health professional education reform for the 21st century: implications of an expert review

**DOI:** 10.1186/s12960-016-0109-8

**Published:** 2016-04-12

**Authors:** Constance Newman, Crystal Ng, Sara Pacqué-Margolis, Diana Frymus

**Affiliations:** IntraHealth International, 6340 Quadrangle Drive, Suite 200, Chapel Hill, NC 27517 USA; United States Agency for International Development, 2100 Crystal Drive, Arlington, VA 22202 USA

**Keywords:** Gender, Transformative, Sexual harassment, Health workforce, Health professional education, Education reform, Discrimination, Health systems

## Abstract

**Background:**

Gender discrimination and inequality in health professional education (HPE) affect students and faculty and hinder production of the robust health workforces needed to meet health and development goals, yet HPE reformers pay scant attention to these gender barriers. Gender equality must be a core value and professional practice competency for all actors in HPE and health employment systems.

**Methods:**

Peer-review and non-peer-review literature previously identified in a review of the literature identified interventions to counter gender discrimination and inequality in HPE and tertiary education systems in North America and the Caribbean; West, East, and Southern Africa; Asia; the Middle East and North Africa; Europe; Australia; and South America. An assessment considered 51 interventions addressing sexual harassment (18), caregiver discrimination (27), and gender equality (6). Reviewers with expertise in gender and health system strengthening rated and ranked interventions according to six gender-transformative criteria.

**Results:**

Thirteen interventions were considered to have transformational potential to address gender-related obstacles to entry, retention, career progression, and graduation in HPE, when implemented in core sets of interventions. The review identified one set with potential to counter sexual harassment in HPE and two sets to counter caregiver discrimination. Gender centers and equal employment opportunity units are structural interventions that can address multiple forms of gender discrimination and inequality.

**Conclusions:**

The paper’s broad aim is to encourage HPE leaders to make gender-transformative reforms in the current way of doing business and commit to themselves to countering gender discrimination and inequality. Interventions to counter gender discrimination should be seen as integral parts of institutional and instructional reforms and essential investments to scale up quality HPE and recruit and retain health workers in the systems that educate and employ them. Implementation challenges spanning financial, informational, and cultural barriers need consideration. The application of core sets of interventions and a strong learning agenda should be part of ongoing HPE reform efforts.

## Background

### Introduction

With the Sustainable Development Goals (SDGs), the global community sees greater than ever consensus about the crucial role that the health workforce plays in realizing goals to achieve universal health coverage [1, 2]. Qualified health workers, trained to work in effective teams within and across professional cadres to address the biomedical and social determinants of health, are critical to achieving health goals. While great progress has been made in maternal and child health and HIV/AIDS, continued shortages of adequately trained health workers raise the question of whether health professional education (HPE) systems are producing the health workforce needed to meet outstanding and emerging global health challenges.

In 2010, the independent Commission on Education of Health Professionals for the 21st Century (henceforth “the independent Commission”) released a comprehensive report on HPE. The Commission’s report spurred a growing movement for HPE reform in many regions, including Africa, Asia, and the Americas [3]. The report calls for institutional and instructional HPE reforms to target a variety of systemic problems [3], including:

- Outdated, fragmented, and content-oriented curricula that produce graduates with narrow contextual understanding and insufficient knowledge, skills, and competencies to understand social and other determinants of health and disease
- Poor teamwork and inadequate collaboration within and across health professional cadres
- Episodic encounters with patient illnesses rather than continuous and holistic health care
- A predominant hospital orientation at the expense of primary care
- An imbalance between health workforces and health needs
- Weak leadership in improving health system performance

The independent Commission’s report also mentions persistent gender stratification of professional status as a systemic deficiency and puts forth as one of its proposed reforms the need to pay particular attention to ensuring equal opportunities through more flexible working arrangements and career paths that accommodate temporary breaks, actively addressing gender discrimination and subordination [3]. Yet gender-related deficiencies in HPE are not limited to gender stratification and include other forms of often invisible gender discrimination and inequalities as described in Table 1. This paper identifies how particular gender-related deficiencies in HPE can be addressed as part of both instructional and institutional governance reforms. The definitions of the terms used in the paper are in Table 2.

**Table 1:** Gender discrimination in health workforce systems [4]

Form of discrimination	Description
Sexual harassment	Unwanted, unwelcome, or offensive conduct that changes the terms and conditions of school or work, where either a person’s rejection of, or submission to, such conduct is used explicitly or implicitly as a basis for a decision that affects that person’s education or career (quid pro quo), or conduct that creates an intimidating, hostile, or humiliating work environment for the recipient (hostile environment). A form of violence as well as discrimination.
Pregnancy discrimination	Exclusions, restrictions, or distinctions made on the basis of pregnancy, childbirth, or related conditions, such as unwillingness to hire, promote, or retain female students or workers who may get pregnant and leave school or the workforce or who require maternity leave and benefits. This type of discrimination is related to:
Family responsibility discrimination	Exclusions, restrictions, or distinctions against individuals (such as pregnant women, mothers and fathers of young children, parents of disabled children, and individuals who care for their aging parents or sick spouses/partners) based on their responsibilities to care for family members. Pregnancy and family responsibility discrimination are related forms that target a broad range of reproductive functions and may be viewed together as *caregiver* (*or reproductive role*) *discrimination*. These related forms of discrimination usually target women of childbearing age who are not able to equally access opportunities for education, hiring, or promotion.
Occupational gender segregation	Concentration of men and women in different jobs (horizontal) or at different levels (vertical) in a job hierarchy. What has been called gender stratification [3] may refer to vertical or horizontal segregation or both.
Gender stereotyping	Overgeneralized characterizations of persons in a particular group, occurring when the personal characteristics deemed necessary for a job are inconsistent with characteristics generally associated with a particular sex.

**Table 2:** Key definitions

*Bias* [43] An inclination or prejudice for or against one person or group, especially in a way considered to be unfair, that often results in discrimination.
*Discrimination in employment and occupation* [33] Practices that place individuals in a subordinate or disadvantaged position in school, the workplace, or the labor market because of characteristics (e.g., race, sex, age, religion, or other attribute) that bear no relation to the person’s competencies or the inherent requirements of the job. Discrimination occurs when bias is enacted.
*Equal opportunity and non-discrimination* [44] The offering of employment, pay, or promotion to all, without discrimination as to sex, race, color, disability, and so forth.
*Gender-blind* [23] Gender-blind policies and programs that are designed without prior analysis of culturally defined economic, social, and political roles, responsibilities, rights, entitlements, obligations, and power relations associated with being female and male and the dynamics between and among men and women, boys and girls. Gender-blind policies and programs ignore gender considerations altogether.
*Gender discrimination* [45] Any distinction, exclusion, or restriction made on the basis of socially constructed gender roles and norms that prevents a person from enjoying full human rights.
*Gender equality* (*in the health workforce*) [46] A condition where women and men can enter the health occupation of their choice, develop the requisite skills and knowledge, be fairly paid, enjoy fair and safe working conditions, and advance in a career, without reference to gender. It implies that health professional education schools and workplaces are structured to integrate family and work to reflect the value of caregiving for women and men.
*Gender inequality* Denotes the gender-based differences that result from gender discrimination and serve to diminish or enhance individuals’ opportunities, access, power, conditions, and/or income.
*Gender transformative* [23] Policies and programs that seek to transform gender relations to promote equality and achieve program objectives. This approach attempts to promote gender equality by (1) fostering critical examination of inequalities and gender roles, norms, and dynamics; (2) recognizing and strengthening positive norms that support equality and an enabling environment; and (3) promoting the relative position of women, girls, and marginalized groups and transforming the underlying social structures, policies, and broadly held social norms that perpetuate gender inequalities.
*Special measures* [19] Programs, policies, and laws that seek to neutralize and redress embedded structures of discrimination and preferences for privileged groups that are already built into social institutions. Such affirmative measures place women or other marginalized groups in a situation of comparative advantage for a limited period, with the aim of achieving substantive equality in the long term.
*Substantive equality* [19] Takes into account the effects of past discrimination and recognizes that rights, entitlements, opportunities, and access are not equally distributed throughout society and therefore the need to sometimes treat people differently (through special measures) to achieve equal results.
*Systemic structural discrimination* [19] Patterns of behavior, policies or practices, and social, economic, or cultural background conditions that are part of the structures of institutions, which create or perpetuate disadvantage for members of a marginalized group relative to other groups in society or organizations.
*Intersectionality* A feminist theory and analytical tool for understanding and responding to the ways in which gender intersects with other identities. The experiences of marginalization and privilege are not only defined by gender but by other identity factors, such as race, class, and sexual orientation to name a few—all of which are determined, shaped by, and imbedded in social systems of power. Intersectional paradigms view race, class, gender sexuality, and ethnicity among others as mutually constructing systems of power ([47, 48]; references 36, 49–52 present the theoretical and methodological issues and opportunities related to this theory).

Gender discrimination—whether culturally, socially, or structurally driven—is sometimes evident in health worker education and employment systems and sometimes so normative as to be invisible. Where gender discrimination and inequality exist, they can hinder production of the robust and competent health workforces needed to achieve health and development goals, by subjecting students and faculty to gender-based exclusions or restrictions and diminishing access and opportunities. As systemic problems that stem from (among other things) institutional cultures, norms, and policies, gender discrimination and inequality should be addressed in HPE reforms.

To date, however, the HPE reform movement has paid scant attention to gender discrimination toward and among students and faculty. The new SDGs include a standalone goal that aims for achieving gender equality and empowering all women and girls, including a target to end all forms of discrimination against all women and girls [1]. This gives gender discrimination and inequality a new primacy in development policy.

As the independent Commission’s report emphasizes, a key outcome of educational reform should be transformative learning [3], which can cultivate a “new professionalism” and establish “enlightened change agents” who have the status, authority, ability, and willingness to challenge the numerous structural and cultural factors that keep gender-based discrimination in place. Transformative learning to address gender discrimination must necessarily be gender transformative. Equal opportunity, non-discrimination, gender equality, and respect for human rights should be core health professional values and competencies, promoted and enacted by HPE leaders, embedded in curricula, and enacted through HPE instruction, professional socialization, and institutional governance as foundations for the professional practice of future health workforce managers and frontline service providers.

This paper summarizes the findings of an expert review that sought to assess interventions to combat gender discrimination and inequality in HPE settings and rank them according to whether they counter two broad types of gender discrimination in transformative ways: discrimination based on pregnancy and on family responsibilities (hereinafter called “caregiver discrimination”) and sexual harassment. We also discuss challenges to implementing the identified interventions, recommendations for addressing the challenges, and implications for HPE reform.

### Gender discrimination and inequality

The global literature on gender and human resources for health (HRH) has demonstrated that gender discrimination and inequality are key barriers to entry, reentry, and retention in employment systems, especially for female health workers [4–6]. The common forms of gender discrimination documented in health workforce employment systems (Table 1) also appear to operate in HPE settings, affecting HPE students’ opportunities, treatment, and ability to complete their studies (Table 3), and limiting faculty members’ career satisfaction, advancement, and economic opportunities (Table 4). We focus on sexual harassment and caregiver discrimination because they were apparent from the interventions described in the literature and because they are relevant to female HPE students and faculty [7].

**Table 3:** Students’ experience of gender discrimination and inequalities

Phase of academic life cycle (students)	Examples and results
Career selection	• Gender stereotypes and segregation in health professional cadres such as nursing and nutrition (Kenya) [10]
Admission and entry	• Negative attitudes against girls and women pursuing training and scholarship opportunities [6] • Lower admission rates of female students at tertiary education institutions (Rwanda) [53]
Course participation and completion	• Threats of failing grades against female students made by male faculty if students refuse sexual advances, leading to difficulty concentrating on studies or failed courses (Ghana, Kenya, Nigeria, Uganda, Zimbabwe) [10, 20, 54–56] • Demotion fees levied against students for taking time off for pregnancy and falling behind in their programs (Kenya) [10]
Career progression	• Sexual harassment during medical training, affecting selection of medical specialty and residency programs (Japan, Sweden, USA) [5, 57–60] • Attitudes discouraging female medical residents from becoming pregnant (USA) [8]
Retention and graduation	• Unsafe living conditions, limiting students’ ability to safely access university facilities and contributing to dropouts of female students [11] • Threats of failing grades against female students by male faculty if students refuse sexual advances, leading to graduation delays or dropouts (Ghana, Kenya, Nigeria, Uganda, Zimbabwe) [10, 20, 53–56] • Required suspension or termination of studies when female students become pregnant (Namibia, Kenya) [9, 10] • Insufficient time for students with family and domestic responsibilities to participate in educational opportunities, contributing to dropouts (Tanzania, Uganda, UK, USA) [11–13] • Higher dropout rates of female students at all educational levels (Rwanda) [53]

**Table 4:** Faculty members’ experience of gender discrimination and inequalities

Phase of academic life cycle (faculty)	Examples and results
Recruitment	• Challenges in balancing work and family obligations, contributing to faculty leaving their positions or turning down employment offers (USA) [31] • Hiring preferences for male faculty due to belief that female faculty taking maternity leave will be disruptive (Kenya) [10]
Career advancement opportunities	• Discriminatory promotion decisions against female staff due to resistance to sexual advances (Nigeria) [20] • Lower number of publications by female medical faculty with children (USA) [31] • Taking reduced workload or time off for family responsibilities seen as a reflection of lower commitment to work (Australia, USA) [16, 61]
Leadership	• Higher numbers of male faculty in senior leadership, even in cadres traditionally considered female occupations, such as nursing (Kenya) [10]
Satisfaction and retention	• Lower rates of career satisfaction among female medical faculty with children than among male medical faculty with children (USA) [15, 17]

#### Students

For HPE students, gender discrimination and inequalities are apparent at different points throughout educational careers, from admission to career track designation to graduation. At the outset of the educational continuum, cultural and gender norms and stereotypes related to childbearing and childrearing can discourage girls and women from pursuing HPE training and scholarship opportunities [6]. Once students are enrolled, gender-blind institutional policies and practices (i.e., policies and practices that do not take gender considerations into account) may prevent female HPE students from participating in classes, practicums, and other curricular offerings by failing to consider potential conflicts between educational requirements and students’ caregiving responsibilities. Such gender blindness typically results in a lack of instrumental support for students, creating barriers to students’ ability to equally access education or remain enrolled. Some professional programs also discourage women from becoming pregnant while they are students [8]. In several countries, pregnant students are required to take mandatory time off before returning to school or may even face expulsion [9]. Caregiver discrimination also may play out in the form of demotion fees for pregnant students who take time off and fall behind in their courses and practicums [10]. Caregiving responsibilities have been shown to play a major role in attrition rates in countries such as Kenya, Tanzania, Uganda, the UK, and the USA [10–13].

In higher-education systems as a whole, insufficient and sometimes insecure living conditions can limit female students’ ability to safely access university facilities and further contribute to decisions to drop out [11]. Sexual harassment and assault have been documented in primary and secondary schools and universities in high- and low-resource settings [14]. For HPE students, sexual harassment, threats, or assault by other students or teachers (whether quid pro quo or in a hostile environment) can make it difficult for the targeted student to concentrate on or complete coursework [10]. Sexual harassment can fundamentally change students’ educational environment and opportunities and may contribute to a student’s decision not to pursue a particular career track.

#### Faculty

For faculty, gender discrimination and associated inequalities often relate to conditions that structurally disadvantage members of one sex (typically women) in the academic system, such as requiring training that involves travel in order to obtain promotions [6]. An academic culture of long working hours and implicit biases against faculty with family responsibilities can affect promotion and tenure decisions in both HPE and general higher-education institutions [15–17]. Recent organizational research found that pregnant women were perceived as less competent, less committed to their jobs, and furthest from meeting male “ideal worker” norms [18]. Such forms of invisible bias and structures of discrimination can be embedded in social institutions over time and become culturally normative [19].

Sexual harassment of faculty members can also affect career advancement. For example, Nigerian female academic staff reported that their refusal of university officials’ sexual advances led to exclusion from promotion and other benefits [20]. This type of discrimination lowers faculty self-confidence, career satisfaction, and retention, which in turn can affect the quality of education being provided at HPE institutions and contribute to faculty attrition [5]. HPE institutions may enable (and fail to regulate) sexual harassment due to cultural norms that tolerate harassment and gender-blind policies [21]. The failure to counter sexual harassment in HPE systems can have a serious and detrimental impact on both student and faculty experiences, as seen by system dysfunctions such as sex in exchange for grades or academic career advancement [20].

The following sections describe our review of interventions to counter caregiver discrimination and sexual harassment in high- and low-resource school-based settings.

## Methods

### Review and assessment of gender-transformative potential

**Fig. 1:**
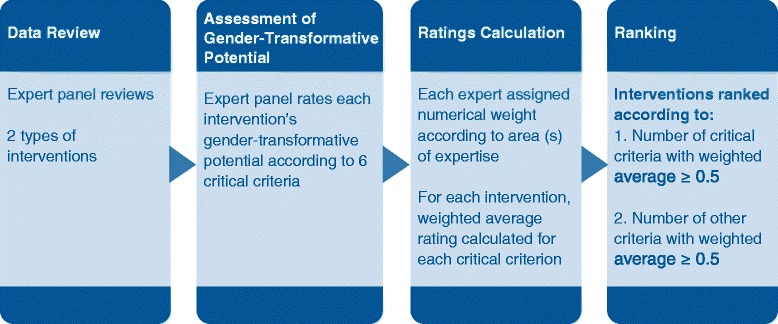
Stages in the review, rating, and ranking of interventions

A panel of experts with expertise in gender and health system strengthening reviewed and rated 52 distinct school-related interventions to address sexual harassment and caregiver discrimination in high- and low-resource settings (Fig. 1).

There were 87 distinct educational institutions/organizations/programs that were included in the literature review, most of which were universities. Of the 87 institutions, the geographical breakdown was:

- North America and the Caribbean, 33 (38 %)
- West, East, and Southern Africa, 32 (36.8 %)
- Asia, 10 (11.5 %)
- Middle East and North Africa, 5 (5.75 %)
- Europe, 4 (4.6 %)
- Australia, 2 (2.3 %)
- South America, 1 (1.2 %)

Interventions that had been previously identified in a review of 379 articles from peer-review and non-peer-review literature on HPE and general tertiary education [22] provided the initial pool of interventions. The experts then assessed interventions’ gender-transformative potential using criteria described in Table 5 by a given type of discrimination, focusing on key aspects such as location(s) where implemented, intervention features, and results of any formal evaluations or informal assessments. One practice was subsequently removed from the analysis because there was not enough information about the intervention itself, resulting in a final count of 51 interventions: 18 to counter sexual harassment, 27 to counter caregiver discrimination, and 6 to address gender equality more generally. Detailed, contextual information related to the review and assessment of interventions is available [22].

**Table 5:** Criteria used to rate gender-transformative interventions in health professional education

Criteria	Sexual harassment
Take measures to end impunity for perpetrators of sexual harassment and other forms of gender discrimination	Top critical criterion to counter sexual harassment
Introduce, make use of, or further legal protections against gender discrimination	Second critical criterion to counter sexual harassment
Provide information and education about discrimination or rights	Third critical criterion to counter sexual harassment
Criteria	Caregiver Discrimination
Transform family, school, and/or work arrangements so that women are not penalized or disadvantaged for caregiving	Top critical criterion to counter caregiver discrimination
Challenge and change common discriminatory gender beliefs or norms	Second critical criterion to counter caregiver discrimination
Attempt to change imbalance of power or otherwise level the playing field	Third critical criterion to counter caregiver discrimination

Most interventions lacked the type of evaluation data related to inputs, processes, and outcomes that would allow the reviewers to determine their effectiveness, feasibility, or sustainability. Thus, reviewers rated the interventions’ *potential* to counter gender discrimination and inequality by applying the six gender-transformative criteria in Table 5. These criteria were formulated by the review team based on the USAID-supported Interagency Gender Working Group definition of gender-transformative policies and programs (see Table 2) [23, 24]. The criteria were considered to be minimum standards for classification of a gender-transformative intervention in HPE settings. The reviewers discussed the meaning of the criteria and marked “Yes” or “No” for each criterion for each intervention to be rated.

### Intervention rating and ranking

For interventions targeting the two types of discrimination (i.e., sexual harassment and caregiver discrimination), each reviewer was assigned a weight based on the reviewer’s area of expertise, with assigned weights totaling 100 %. For each of the 51 practices, reviewers rated interventions applying the six criteria, with 0 representing “No” and 1 representing “Yes.” The researchers then derived a weighted average for each intervention. The expert panel decided that an intervention had at least some gender-transformative potential if its weighted average was 0.5 or above. To rank interventions, one that had a 0.5 rating would be ranked higher than one with a 0 rating. Interventions were ranked by taking inventory of the reviewers’ ratings using these critical criteria [22].

The reviewers met to discuss the ranking process and final rankings. During these meetings, they refined their application of the six gender-transformative criteria, developed recommendations about core sets of interventions, and developed cross-cutting recommendations related to countering the two types of discrimination.

## Results

### Core sets of intervention

Of the 51 interventions reviewed, 13 interventions were identified as having significant stand-alone transformative potential in terms of the transformative criteria (see Table 5) and more so when implemented in combination, that is, in “core sets.” The reviewers formulated these core sets to include those interventions that were necessary (though not sufficient) to counter a particular form of discrimination in HPE settings. The core sets of priority interventions to counter sexual harassment and caregiver discrimination were those that met the critical criteria such that an intervention that met, for example, the top two critical criteria was ranked higher than an intervention that met only the top critical criterion. The panel also identified implementation challenges for each set where such information was available and formulated recommendations for addressing those challenges.

#### Core set to counter sexual harassment

The expert reviewers identified a core set of three gender-transformative interventions with the potential to counter sexual harassment in HPE, shown in Table 6. Establishing a sexual harassment policy and a grievance procedure appears to be feasible across high- and low-resource settings, as evidenced by implementation of the two practices in a number of African and North American universities [22].

**Table 6:** Interventions included in the core set to counter sexual harassment in health professional education

Intervention	Description
Sexual harassment policy	• Includes a single code of conduct for students, faculty, and staff
Grievance or reporting procedure	• Is confidential, outlines consequences for perpetrators, and takes concrete action to end impunity and reduce victims’ fear of or vulnerability to retribution
Education and awareness raising	• For students, faculty, and staff

#### Key implementation challenges for interventions to counter sexual harassment

Although many HPE and other higher-education institutions included in the review had implemented one or more of the interventions in the core set to counter sexual harassment, the review identified a number of challenges:

- Sexual harassment policies may outline strong principles and institutional responsibilities, but the practical implementation of such policies can differ widely from their intentions. For example, the University of Stellenbosch’s (South Africa) policy mandates a sexual harassment advisory and disciplinary committee, yet an assessment found that not only were many managers unaware of the policy but committee members’ workloads made trainings on the policy difficult to schedule and implement [25].
- The lack of awareness of grievance procedures (and of sexual harassment policies), along with inadequate individual and institutional training, can contribute to anemic use of grievance procedures in settings where sexual harassment is normative.
- Most policies explicitly prohibit retaliation against victims who report sexual harassment, but flawed grievance procedures and prevailing environments of intimidation or impunity can render anti-retaliation policies ineffective.
- Fear of retribution and lack of accountability discourage many victims of sexual harassment from using grievance procedures [25–28]. Assessments of Chancellor College in Malawi [26] and the University of Botswana [27] noted that when cases were reported, significant errors occurred in handling investigations, maintaining confidentiality, assuring that alleged harassers showed up, coordinating with responsible agencies, and even following the prescribed procedures, which caused students to lose confidence in the process.

Given the possibility of culturally normative and unregulated sexual harassment in HPE settings, grievance procedures are an important intervention. To address the identified challenges, grievance procedures should pay special attention to confidentiality, guidance for documenting and reporting, clearly outline consequences for the perpetration of sexual harassment and retaliation, avoid an inadvertent chilling effect on reporting that may result from an overemphasis on false reporting, and take concrete action to both decrease and eliminate fear of retribution. Equally important, strategies must be implemented and enforced through strong institutional leadership, vigilant oversight, and timely follow-up and resolution.

#### Core set to counter discrimination based on caregiver responsibilities

The reviewers also identified two core sets of interventions for students and faculty to counter caregiver discrimination (Table 7). Practices included in these core sets have been shown to be feasible in some settings, with institutions in South Africa, Tanzania, and other countries offering child care [29, 30]. However, of the institutions reviewed, only the University of California and the University of Michigan, both in North America, offered the full set of interventions comprising the core set for faculty, and no institutions were identified that offered the full core set for students [22].

**Table 7:** Interventions included in the core sets for HPE students and faculty to counter caregiver discrimination

	Core set for students	Core set for faculty
Pregnancy	• Pregnancy/maternity and parental leave	• Pregnancy/maternity and parental leave (paid)
	• Continuation and reentry policies that do not require pregnant students to terminate their education	• Pregnancy/maternity leave replacement funding to hire temporary replacements for employees on pregnancy/maternity leave to ensure continuity of instruction
Postpartum	• Lactation breaks and spaces	• Lactation breaks (paid) and spaces
	• Parental leave	• Parental leave
	• Child care (daily and emergency)	• Child care (daily and emergency)
	• Child care financial assistance (or at low cost)	• Child care financial assistance (or at low cost)
	• Flexible training schedules, such as part-time schedules and reduced workloads	• Flexible working hours • Flexible tenure

#### Key implementation challenges for interventions to counter caregiver discrimination

Interventions to counter discrimination based on caregiver responsibilities also face implementation challenges, although these, too, can be met by strong HPE leadership commitment:

- Adverse consequences—or fear thereof—are a significant barrier associated with some interventions. For example, faculty who opt for reduced duty leave or flexible training programs may experience resentment from colleagues. Moreover, HPE faculty may not always take advantage of interventions for fear that others will perceive them as uncommitted or that their careers will be negatively affected.
- Work-life integration is a key concern for many current and prospective HPE faculty (both women and men) [31]. Institutions with family-friendly policies may, therefore, have a competitive edge in recruitment. Indeed, outside of the HPE sector, the University of Washington law school has used its family-friendly environment as a student- and faculty-recruiting tool [32], and the University of California and University of Michigan both highlight their family-friendly initiatives to faculty candidates.
- Families and communities may resist some of the changes required to address discrimination based on caregiver responsibilities, because the interventions challenge longstanding gender norms, expectations, and divisions of labor. Girls and women who go to school likely need a reduced workload at home, potentially adding to their families’ workload.

Communication of policy and education of faculty and students, as well as ongoing public support of faculty who use such flexible policies, is key to preventing adverse consequences. A complementary strategy is to proactively plan for pregnancy coverage and flexible scheduling.

HPE planners must also anticipate the different levels of resistance that may arise in recruitment and retention efforts and deliberately mobilize communities around reducing women’s and girls’ housework, preventing early marriage and pregnancy and sharing responsibility for caregiving. This implies a long-term, multidimensional, and multisectoral strategy to keep girls in school from the primary through tertiary levels. This might include provision of reproductive health services (including family planning) through HPE institutions.

#### Interventions that address multiple forms of gender discrimination and inequality

In addition to selecting the core sets of interventions targeting sexual harassment and caregiver discrimination, the reviewers identified gender centers and equal employment opportunity units as having significant gender-transformative potential. These are institutional structures that advocate for, coordinate, oversee, implement, and evaluate multilevel strategies. These entities generally work to:

- Develop gender equality, equal opportunity, or affirmative action policies
- Engage in awareness raising and information sharing
- Serve advocacy and accountability functions
- Conduct gender sensitization workshops or sexual harassment training for women and men
- Conduct research and university assessments
- Provide financial assistance to female students
- Offer mentoring and faculty career and leadership programs to women

The last two objectives are examples of special measures to counter systemic structural discrimination and promote substantive equality (see Table 2). These special measures counter the discrimination that may occur when poor families allocate scarce financial resources to fund boys’ education and to compensate for the career barriers faced by women in HPE institutions characterized by high concentrations of men in top faculty and administrative positions.

Whereas equal employment opportunity units which aim to counter discrimination in employment and occupation [33] are often backed by national equal opportunity laws, gender centers face the challenge of not necessarily being backed by law. In addition, implementation challenges may arise from funding or staffing constraints. Leaders in HPE reform should educate stakeholders and advocate for the need for resource allocations to fund special measures to counter systemic structural discrimination to achieve substantive equality.

### Research limitations

The relative lack of descriptive contextual and evaluation data for the 51 interventions limited the expert reviewers’ ability to determine the exact nature, feasibility, sustainability, or effectiveness of the various interventions and, therefore, constrained their capacity to make recommendations for specific contexts such as low-resource settings. Overall, more information was available for institutions in high-resource than low-resource settings.

The research team invited a variety of published experts representing different sectors and countries to participate as reviewers, but most experts were unavailable. A larger expert review group (including stakeholders such as students and faculty), or one with a more diverse range of expertise, would likely strengthen future reviews.

## Discussion

A comprehensive HPE reform agenda aiming to produce a robust and competent health workforce should consider core sets of interventions to counter gender discrimination and inequality—even when discrimination is not overtly recognized by perpetrators or victims. Female health workers already constitute a large proportion of many countries’ health workforces (in both the professional and non-professionalized cadres), and there is a growing focus on the role of women in the health workforce in the emerging human resources for health agenda [5]. Failure to address gender discrimination and inequality in HPE can jeopardize broader health workforce and health system reform.

The review findings can serve as a basis for evidence-based decisionmaking in planning and implementing appropriate gender-transformative interventions. The review demonstrated that interventions can address some of the gender-related obstacles to entry, retention, career progression, and graduation in HPE, in particular those related to sexual harassment and caregiver discrimination. Although many HPE institutions may not have the resources to implement *all* the gender-transformative interventions identified by the reviewers, the “core sets” provide a basis for establishing priorities and taking steps to counter gender discrimination. Potential implementation challenges spanning financial, informational, and cultural barriers need to be considered, however. Financing considerations are particularly relevant when interventions entail restructuring physical resources and human resources arrangements (such as child care, lactation spaces, or reduced duties leave) or require human resources (e.g., education and awareness-raising activities or adequately staffed and trained sexual harassment committees).

Merely offering interventions does not guarantee that interventions are used or that equal opportunities and gender equality in HPE will result. Ensuring that the institutional community and especially the intended beneficiaries are aware of, committed to, and actually use the interventions is equally important. However, because many of the interventions challenge social and institutional norms and cultural stereotypes, some community members (inside or outside the institution) may resist their implementation. Increasing the actual use of new policies or procedures requires information and advocacy, anticipating potential resistance, providing incentives, and ensuring that HPE leaders are accountable for work-life integration and the speedy and effective handling of sexual harassment so that institutional efforts actually prevent and end impunity for discriminatory organizational structures and behaviors.

What are some implications for HPE reform? First, gender-transformational HPE reform will in some cases entail both institutional and instructional changes [3] sometimes in combination, since some reforms to institutional governance may require, or can be enhanced by, instructional reforms. For example, the gendering of the health workforce involves the distinction between occupations, as well as the relations between occupations (for instance, between medicine, nursing, and allied health professions) [34]. These processes of gender segregation or stratification could be mitigated by both equal opportunity policy (an institutional governance reform) and transprofessional education (an instructional reform).

Institutional reform, such as introducing a new policy, could also be combined with professional instruction to raise students’ and faculty awareness of gender discrimination as *unethical* professional conduct, as well as a human rights violation. For example, the problem of *quid pro quo* sexual harassment (i.e., sex in exchange for grades, see Table 3) is a form of unethical professional conduct within an HPE, an abuse of power by faculty which impacts educational or occupational opportunity, which has both instructional and institutional consequences. Heads of HPE institutions can work with an equal opportunity unit to introduce a code of conduct to faculty and students and include the subject in a course in ethics or human rights [35], thus playing a dynamic role in inculcating shared attitudes, values, and behaviors related to respect for the dignity and rights of clients, students, and colleagues. Instructors can be trained to model ethical one-on-one and team interactions and conducting fair assessments free from the taint of sexual harassment, stereotyping, and other forms of discrimination.

Third, the review highlights the importance of a gender-relational perspective in HPE reform. Gender relations shape health systems—including HPE systems—through their effects on the occupational segregation and stratification among health providers, the conditions of work, and processes of regulation, supervision, and management of health labor forces [36]. The theoretical foundations of the approach in this paper include gender-relational theory, social dominance theory, and the sociology of patriarchy [34, 37, 38]. This perspective gives a central place to the patterned relations between women and men (and among women and among men) that constitute gender as a social structure [39]. The structure of gender relations (including power relations and hierarchies) in a given institution is its “gender regime” [34]. The institutions through which health care is delivered (e.g., hospitals, clinics, private practices) have well-defined gender regimes [34], and this applies to HPE institutions. If so, heads of HPE schools would benefit from a gender analysis approach to shed light on the “gender regime” in their institutions and possible impacts on faculty and students at various points in the academic career. A practical first step would be for heads of schools, equal opportunity staff, or human resources managers to conduct a gender audit of its institutional “gender regime,” including policies and practices in relation to pregnancy, family responsibilities and sexual harassment, instructional content, socialization processes, and the socioeconomic characteristics of its student body and faculty. Ending all forms of discrimination against all women and girls everywhere (i.e., SDG 5.1) does not speak to the multiple or intersecting axes of gender discrimination, bias, and marginalization (e.g.,, economic class, region, race, or caste) which likely impact present and future student bodies. Such analysis could result in changes in recruitment, admissions, and financing [3, 40, 41]. Operationally, this is a “field waiting for an analytical breakthrough” ([36, p. 9]) and should be part of an HPE-learning agenda.

Fourth, institutional governance reforms suggest the utility of gender centers or equal employment opportunity units to drive policy and accountability. This unit could coordinate the development and enforcement of supportive, evidence-based policies to promote equal opportunity, non-harassment, and social equality. As a part of policies such as flexible working arrangements and career paths that accommodate temporary breaks, HPE leaders and HR managers should embrace gender equality in social roles and promote the value of caring and work-life integration *for both women and men* in educational and employment systems [3, 42].

Finally, the review highlighted the striking lack of contextual and evaluation documentation for gender-transformative interventions in HPE and the need to invest in a stronger learning agenda in relation to sexual harassment, caregiver discrimination, and, in fact, in all forms of discrimination already documented in health employment systems. We recommend building a rigorous knowledge base as well as evaluating the feasibility, sustainability, and effectiveness of gender-transformative HPE reforms. Table 8 outlines suggested areas of focus for this learning agenda.

**Table 8:** A learning agenda for gender-transformative health professional education reform

Form of discrimination	Suggested elements of learning agenda
Sexual harassment	• Elements and features of grievance procedures that effectively resolve sexual harassment cases and end impunity • Effectiveness of various (HPE) institutional and non-institutional actors in investigating sexual harassment • Role of extra-institutional legal advocacy and redress • Desirability of separate grievance procedures for students, faculty, and staff versus a single procedure
Caregiver discrimination	• Impact of basic bundle on student retention, performance, and graduation • Impact of basic bundle on faculty recruitment, retention, and development • Feasibility and effectiveness of offering free or low-cost family planning for men and women in the basic bundle • Effective community messaging related to the need to reduce women’s and girls’ family and domestic burdens • Factors that contribute to use of family-friendly services by both women and men
Cross-cutting	• Approaches for inter- and transprofessional education to promote equality and teamwork and effectively eliminate gender stereotyping and stratification (e.g., siloes, hierarchical chains of command) • HPE curricula that transmit core professional values and competencies (including human rights, social justice, gender equality, professional ethics and conduct, respectful care, and critical inquiry) and foster health workers as change agents in inequitable systems • Test new and efficient gender audit methodology and information systems to support improved institutional governance policies and monitor progress of reforms, targeting the analysis of gender regimes and intersecting discriminations, including the collection of data for indicators of social position (e.g., gender, income, geographical region, race, caste, disability) • New approaches to teach human rights and ethical professional conduct in combination with HPE governance. • Coordinated efforts across universities to implement special/affirmative measures and enabling conditions such as work and learning design to take parenting and caregiving responsibilities into account for all students and faculty, universal flexible working/study arrangements. • Structural and resource requirements for and impact of equal opportunity units in HPE institutions

## Conclusions

The paper’s broad aim is to encourage HPE leaders—heads of schools, human resources administrators, donors, and other human resources for health (HRH) stakeholders—to make gender-transformative changes in the current way of doing business and commit themselves to countering gender discrimination and inequality in HPE. These affect the functioning, quality, outputs, and outcomes of health professional education. Gender-transformative HPE reforms that eliminate impunity for sexual harassment, promote and protect the educational and labor rights of students and faculty, and develop student and faculty understanding of professional ethics will require changes in mindsets, institutional cultures, and leadership capability and the reframing of health workers as change agents committed to gender equality in often inequitable systems. Greater focus on the application of the core sets of interventions identified in this paper and a strong learning agenda should be part of ongoing HPE reform efforts.
